# Simultaneous Determination of Streptomycin and Oxytetracycline Using a Oracet-Blue/Silver-Nanoparticle/Graphene-Oxide/Modified Screen-Printed Electrode

**DOI:** 10.3390/bios10030023

**Published:** 2020-03-11

**Authors:** Sanaz Akbarzadeh, Habibollah Khajesharifi, Michael Thompson

**Affiliations:** 1 Department of Chemistry, Yasouj University, Yasouj 75918-74831, Iran; akbarzade.sanaz@yahoo.com; 2 Department of Chemistry, University of Toronto, 80 St. George Street, Toronto, ON M5S 3H6, Canada; m.thompson@utoronto.ca; 3 Institute of Biomaterials & Biomedical Engineering, University of Toronto, 164 College Street, Toronto, ON M5S 3G9, Canada

**Keywords:** oracet blue, graphene oxide, streptomycin, oxytetracycline, screen-printed electrode

## Abstract

In this paper, an electrochemical technique is introduced for the determination of streptomycin (STR) in the presence of oxytetracycline (OTC) in milk samples. A novel bifunctional modified screen-printed electrode (SPE) modified with oracet blue, silver nanoparticles, and graphene oxide (OB/SNPs/GO/SPE) was fabricated. The modified electrode plays a catalyzer role for electrooxidation of STR at pH = 7.0 and reveals a facile a separation between the oxidation peaks of STR and OTC. Calculation of kinetic parameters such as the electron transfer coefficient α and the heterogeneous rate constant *k*´ of STR at the OB/SNPs/GO/SPE as 8.1 ± 0.07 cm s^−1^ and 0.32 have been obtained based on the theoretical model of Andrieux and Saveant. A differential pulse voltammetric measurement demonstrates two linear dynamic ranges, 0.4 to 240.0 nM and 240.0 to 720.0 nM and a detection limit of 0.17 nM for STR. The sensitivities of the OB/SNPs/GO/SPE towards the oxidation of STR in the absence and presence of OTC were 2.625 × 10^−1^ and 2.633 × 10^−1^ µA/µM, respectively.

## 1. Introduction

The aminoglycosides are an important group of antibiotics as their main influence is to produce inhibitory effects on the protein production of bacteria. This group of antibiotics displays significant activity toward Gram-positive and Gram-negative bacterial infections via a disaccharide molecule containing a streptidine aminocyclitol moiety. Streptomycin (STR) is the most basic of the aminoglycosides, is an antimicrobial organic base produced by Streptomyces Griseous, and is commonly used both in human and veterinary medicine [1]. In agriculture, it is used to control bacterial and fungal diseases of selected fruits, vegetables, seeds, specialized field crops, ornamental crops, and in ornamental ponds and aquaria to control algae [2].

Oxytetracycline (OTC) is a member of the tetracycline family produced on an industrial basis by Streptomyces Rimosus fermentation [1]. The molecule is a clinically important natural product possessing semisynthetic derivatives which are characterized by a broad spectrum of activity against pathogenic microorganisms. These groups of reagents are bacteriostatic antibiotics that, as for streptomycin, hinder bacterial cell protein formation. Their major application is the control of bacterial infections in humans and animals. They have also been used in conserving harvesting fruits and vegetables and for prevention of the effects of insect pests. 

Veterinary drugs are used widely in domestic animals for the avoidance and treatment of infectious diseases and for raising growth. The indiscriminate application can lead to the contamination of dairy products such as milk, in turn potentially causing various side effects on consumption including fever and nausea, intensive allergic reactions such as photodermatitis, and anaphylaxis. Accordingly, the development of a selective and sensitive method for the accurate determination of residues of veterinary drugs such as STR and OTC in animal food is highly critical. In this connection, appropriate controlling authorities worldwide have enacted maximum residue limits (MRLs) for a number of veterinary drugs that contaminate food and dairy products.

The determination of aminoglycosides conventionally requires the employment of qualitative microbiological methods rather than quantitative procedures [3,4,5,6]. Chemical analysis methods include high-performance liquid chromatography (HPLC) [7], ion pair chromatography [8], agarose gel electrophoresis [9,10], liquid chromatograph(LC) [11], and mass spectrometry [12]. These methods are costly, generally time-consuming, and usually exhibit interferences from other pieces. Thus, achievement of analysis through the use of inexpensive methods would prove to be an attractive alternative. 

Electrochemical techniques have been applied recently to determine antibiotics since they provide a relatively convenient, rapid, and low-cost approach. In terms of electrodes for electrochemical assay, screen-printed electrodes have received increasing attention due to their high sensitivity, rapid response, low detection limit, facile sample injection, and capability to function in microfluidic configuration. STR is oxidized at common electrodes with a significant oxidation overpotential, thus providing a promising methodology to reduce over-voltage effects and enable the determination by chemically-modified electrodes (CMEs) [13]. Modification of electrodes with appropriate electroactive reagents can result in the enhancement of electrochemical biosensors.

Graphene oxide (GO) is a two-dimensional carbon nanomaterial with a large surface area that possesses many oxygen functional groups such as hydroxyl, epoxide and carbonyl groups, which can be easily activated to an SPE configuration. In addition to a large surface area, the combination of GO with SNPs for electrode fabrication offers very high electrical conductivity, high water solubility, and ease of construction via the electrostatic self-assembly technique. [14,15].

**Scheme 1:**
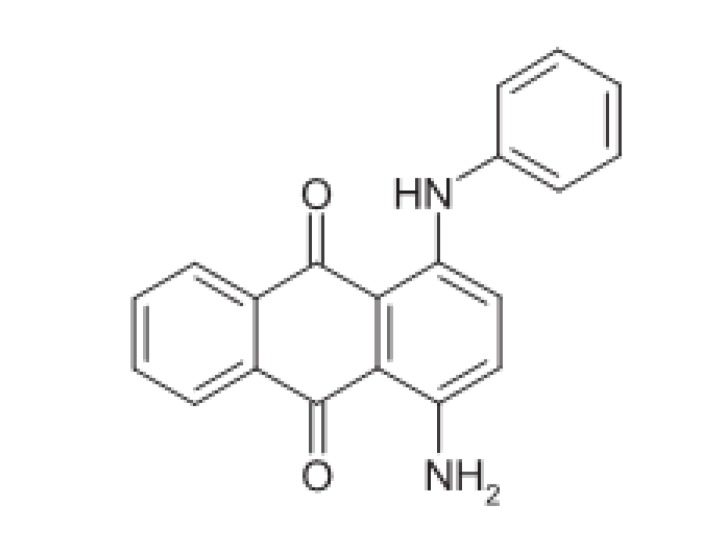
Oracet blue (OB) structure.

The anthraquinone derivative, oracet blue (the structure of which is shown in Scheme 1), can be used as an electrode modifier due to its good reactivity as a mediator through the catalysis of slow reactions. When employed as a modifier, the molecule displays two oxidation peak potentials at 230 and 540 mV (at pH = 7.0). The present work was conducted based on the second peak potential at 540 mV. Based on preparation methods of the OB-modified electrodes reported in the literature [16,17,18], the present work describes the utilization of a oracet blue/silver-nanoparticles/graphen-oxide-modified screen-printed electrode (OB/SNPs/GO/SPE) as an electrocatalyst for the oxidation of STR. Additionally, the successful determination of STR in the presence of OTC in real samples with the prepared modified electrode is described.

## 2. Material and Methods

### 2.1. Reagents and Apparatus

Streptomycin and oxytetracycline and all the chemicals used for the experimental work are of analytical grade purchased from Merck Company. 1-Amino-4-anilinoanthraquinone or oracet blue (OB) also was purchased from Merck (Scheme 1), and graphene oxide (GO) was obtained from Sigma–Aldrich. For the preparation of all the solutions, doubly distilled water was used. Buffer solutions (0.1 mol L^−1^) were prepared from H_3_PO_4_ and adjusted for pH with an NaOH solution. Streptomycin (STR) and oxytetracycline (OTC) solutions were just prepared just prior to use, and all experimental steps were performed at room temperature.

The electrochemical tests performed with an Autolab potentiostat-galvanostat AUT41203 equipped with NOVA 2.1 software (Metrohm Company, Netherland), connected to an SPE through a specific cable connector(CAC)-and for electrochemical measurements a personal computer (PC) system was used. SPEs were purchased from (Dropsense Company, Asturias, Spain model C110) featuring carbon ink as a working electrode, a carbon electrode as counter, and Ag electrode as a reference electrode. The modification of the working electrode (carbon ink) was done to prepare the graphene-oxide-modified SPE (GO/SPE), an OB electrodeposited on a graphene-oxide-modified SPE (OB/GO/SPE), a silver-nanoparticles-modified GO/SPE (SNPs/GO/SPE), and an OB electro-deposited on silver-nanoparticles–Graphene-oxide-modified SPE (OB/SNPs/GO/SPE). The measurement of pH was performed with a Metrohm pH/mV meter (model 827).

### 2.2. Preparation of Samples

As the electrochemical techniques are able to determine the concentration of STR without pretreatment, the preparation of a real sample is produced by diluting 3 mL of fresh milk to 10 mL with a 0.1 M phosphate buffer solution pH = 7.0. Next, the addition of certain amounts of STR was done and the responses of the OB/SNPs/GO/SPE were determined in differential pulse voltammetry (DPV)measurements with the milk samples being directly inserted into the experimental cell. 

### 2.3. Electrode Preparation

**Scheme 2:**
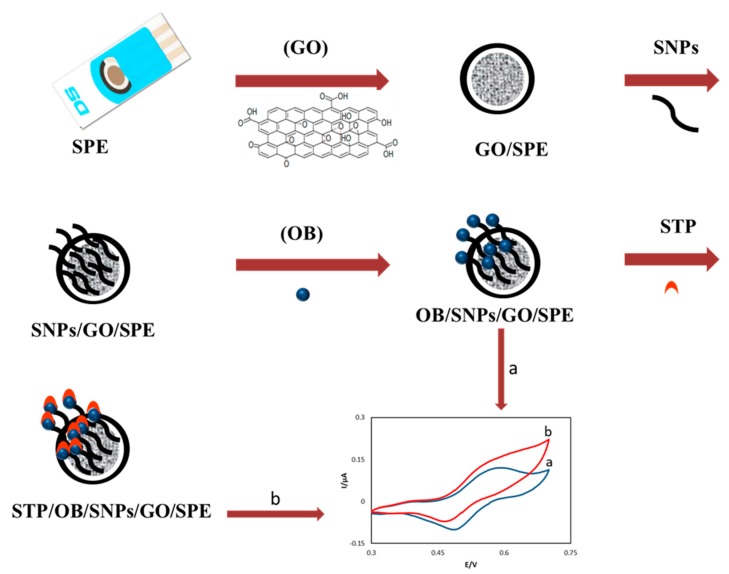
The procedure of electrode preparation.

For the working electrode, a 2.5 µL drop of dispersed (1mg/mL in H_2_O) graphene oxide (GO) was placed on the bare SPE to produce the GO/SPE combination. For the preparation of SNPs/GO/SPE, a 2.5 µL solution of 100 mM nitric acid and 1.0 mM AgNO_3_ was placed on the GO/SPE, and a continuous cyclic potential was performed from −700 to 1900 mV with a scan rate of 80 mV s^−1^ for eight cycles [19]. Following this procedure, the modified electrode was washed with doubly distilled water. The oracet blue SNPs modified GO/SPE (OB/SNPs/GO/SPE) was prepared by placing a 2.5 µL drop of 0.1 mol L^−1^ phosphate buffer pH = 7.0 containing 1.0 mmol L^−1^ of OB, and OB was immobilized on the SNPs/GO/SPE surface by 18 potential cycles from 0.0 to 230 mV at 25 mV s^−1^. To prepare OB modified GO/SPE (OB/GO/SPE), the GO/SPE was modified as explained above without SNPs and lastly doubly distilled water was used to wash the modified electrodes as is shown in Scheme 2.

**Figure 1:**
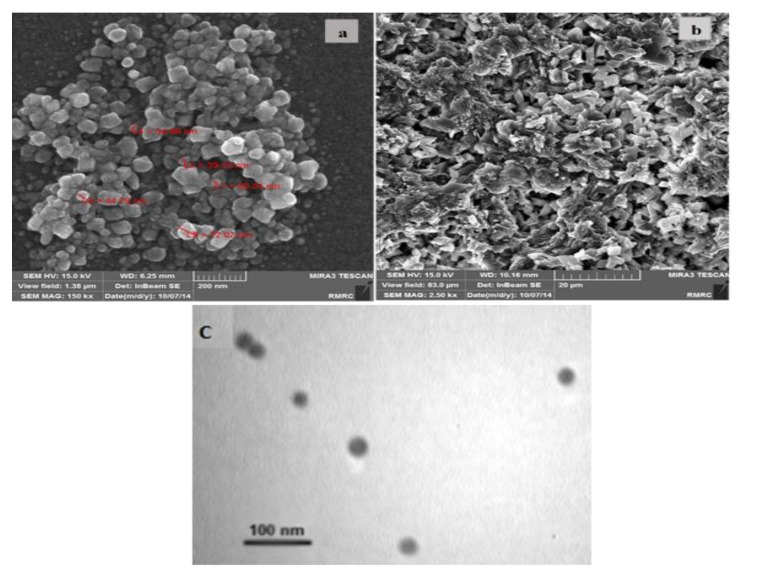
SEM images of the morphology of (**a**) silver-nanoparticles/graphene-oxide-modified screen-printed electrode (SNPs/GO/SPE), (**b**) oracet-blue/silver-nanoparticles/graphene-oxide-modified screen-printed electrode (OB/SNPs/GO/SPE) and (**c**) the TEM image of SNPs.

Given the importance of electrode surface morphology, characterization of the electrodes was assessed by SEM. The morphology of SNPs/GO/SPE and that of OB/SNPs/GO/SPE are shown in Figure 1a,b. Characterization and specification of the SNPs were achieved using transmission electron microscopy (TEM) imaging (Figure 1c). The resulting SNPs have sufficient uniform shape and approximately 30 nm diameter. Figure 1b shows that a thin film of OB was adsorbed on SNPs/GO/SPE. Since, in this work, the SPE surface was covered by SNPs, we believe that the deposition of OB at the SNPs/GO/SPE was mainly due to the strong adsorption of OB onto SNPs/GO/SPE.

## 3. Results

### 3.1. Electrochemical Charaterization of the OB/SNPs/GO/SPE Electrode

**Figure 2:**
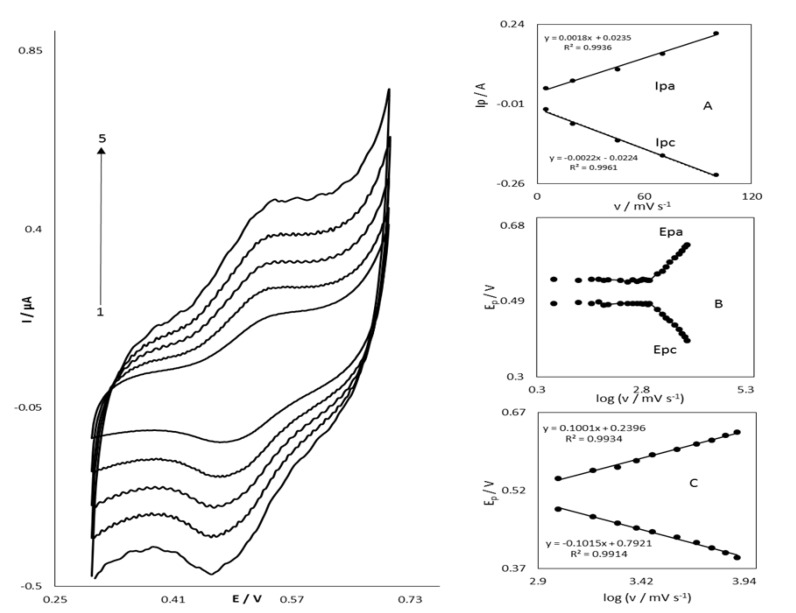
Cyclic voltammetric responses of GO/OB/SNP/SPE in 0.1 M phosphate buffer pH = 7.0 at different scan rates (The numbers 1–5 correspond to 20–100 mV s^−1^). Insets: (**A**) plots of anodic and cathodic peak currents vs. of scan rate. (**B**) Variation of the peak potentials vs. the logarithm of the scan rate. (**C**) Magnification of the same plot for high scan rates.

The cyclic voltammograms of OB/SNPs/GO/SPE in 0.1 mol L^−1^ phosphate buffer pH = 7.0 at several scan rates are presented in Figure 2. An excellent redox couple appears when the potential is applied from 300 to 700 mV without a background current. OB electrodeposited on a screen-printed electrode (SPE) modified with graphene oxide and SNPs (OB/SNPs/GO/SPE) shows a redox couple. Schemes of the anodic and cathodic peak currents vs. the scan rate show a linear relationship (Figure 2, inset A) as estimated for the electrode surface which is immobilized with an electroactive couple. Furthermore, the formal potential (*E_0_´*) value, by the use of the equation of *E_0_* = *E_pa_–α*(*E_pa_* – *E_pc_*) [20] is around 500 mV and for scan rates in the range of 5 to 1000 mV s^−1^ is nearly independent of the scan rate of potential (Figure 2, inset B). As shown in Figure 2, inset C, for scan rates higher than 2000 mV s^−1^, the values of the anodic and cathodic peak potentials are related to the logarithm of the scan rate.

In these circumstances, the surface electron transfer rate constant (k_s_) and the charge transfer coefficient (*α*) for electron transfer between OB and SNPs/GO/SPE can be predicted from the slope of the linear correlation between peak potentials (Ep) and the logarithm of the scan rate as [Slope = 2.3RT/(1–*α*) *n_α_ F*]. This result agrees with the Laviron theory [21]. Based on the theoretical considerations mentioned above, the rate constant of electron transfer in the middle of SNPs/SPE and OB can be determined by the following equation:

*log ks* = *α log(1 − α) + (1 − α)log α − log (RT/nFv) − α(1 − α) nF ∆Ep/2.3RT* (1)

where (1 − *α*) *n_α_* = 1.02, *v* is the scan rate, and all other signs having their usual meanings. A mean of k_s_ = 14.0 ± 0.5 s^−1^ is obtained applying Equation (1) to all experimental data. The *k*_s_ is the constant of electron transfer between SNPs/GO/SPE and immobilized oracet blue on the surface of this modified electrode. The mediator for electron transfer between the modified SPE and STR is oracet blue, which can decrease the overpotential of STR oxidation on the SPE surface. Therefore, the large value of *k*_s_ validates the fast formation of the equilibrium. The obtained value for *k*_s_ and those reported for other modifiers before are in agreement with each other. The value of *k_s_* described in this research was compared with other works [22,23,24,25]. To obtain the kinetic parameters *α_c_* (cathodic transfer coefficients) and *α_a_ = (1 − α_c_* ) (anodic transfer coefficients) of the slopes of Figure 2, plots can be used. The slope of the linear part is −2.303*RT*/*α_c_* F for the cathodic peak and 2.303*RT*/*α_a_* nF for the anodic one. We considered the mean value of 0.49 for *α_c_* (*α*) and used it in the following study. The range of transfer coefficient, *α*, is from zero to one, which indicates how useful is the reduction of a reagent toward its oxidation.

### 3.2. Electrocatalytic Oxidation of STR at the OB/SNPs/GO/SPE

**Figure 3:**
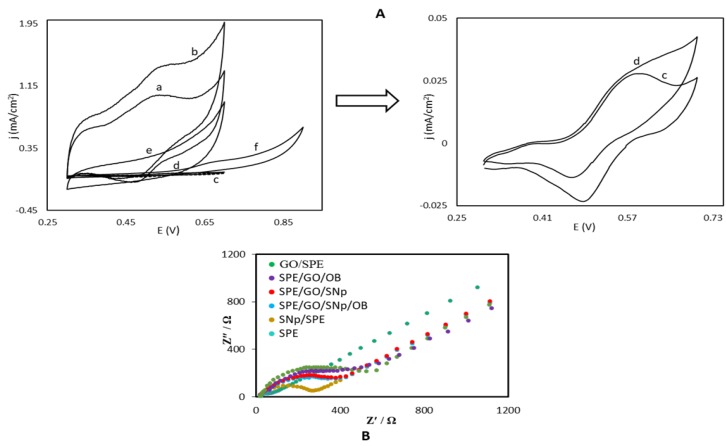
(**A**) Cyclic voltammograms of OB/SNPs/GO/SPE in the absence (a) and presence (b) of 0.1 mM STR. Cyclic voltammograms of SNPs/GO/SPE (c) and OB/GO/SPE (d) in the presence of 0.1mM STR. (e,f). As (b) for SNPs/SPE, and GO/SPE, conditions: 0.1 M phosphate buffer solution pH = 7.0 at scan rate 20 mV·s^−1^. (**B**) Electrochemical impedance spectroscopy of the electrode for SPE, GO/SPE, SNPs/SPE, SNPs/GO/SPE, OB/GO/SPE, and OB/SNPs/GO/SPE.

One of the purposes of this study was the separation of the electrochemical responses of STR and OTC from each other with a modified electrode for electrocatalytic oxidation of both molecules. To examine the electrocatalytic performance of the OB/SNPs/GO/SPE towards the oxidation of STR, Figure 3A presents the CV responses which were measured at pH = 7.0 phosphate buffer solution in the absence and presence of STR. The anodic current density increases noticeably compared to a recorded scan for a buffer solution without STR. The cause of the increase in oxidation current density is the diffusion of STR in the solution to the surface of modified electrode followed by conversion of OB_(ox)_ to OB_(red)_ reduced by STR. As STR regenerates OB (red), an intense increase in the anodic current density appears at the potential scanning duration; similarly, the cathodic peak current density decreases while the STR is present (Figure 3A, curve d). Equations (2) and (3) describe the electrocatalytic oxidation of STR. The reaction of OB with STR is shown in Equation (3) with rate constant *k´* relating to the rate-determining step. Accordingly, the rate of the oxidation of the modified surface with OB in Equation (2) *k_s_* in comparison with the reaction of the OB with STR can be considered fast.

(2) $${\mathrm{Oracet}\;\mathrm{blue}}_{\left(\mathrm{red}\right)}\;\begin{array}{c} k_{s}\\ ⇄ \end{array}\;{\mathrm{Oracet}\;\mathrm{blue}}_{\left(\mathrm{ox}\right)}\;+\;2H^{+}\;+\;2e$$

(3) $${\mathrm{Oracet}\;\mathrm{blue}}_{\left(\mathrm{ox}\right)}\;+\;{\mathrm{STR}}_{\left(\mathrm{red}\right)}\;\overset{K´}{\to }\;{\mathrm{Oracet}\;\mathrm{blue}}_{\left(\mathrm{red}\right)}\;+\;{\mathrm{STR}}_{\left(\mathrm{ox}\right)}$$

The peak potential of anodic segment for the STR oxidation at OB/SNPs/GO/SPE is about 540 mV (Figure 3A, curve d), whereas STR is oxidized at about 670 mV (Figure 3A, curve f) at a GO/SPE under the same conditions and shows a lower peak current density than that seen with OB/SNPs/GO/SPE. Thus, the overpotential decreased for 130 mV, and the peak current was increased by the modified electrode. There is no oxidation peak potential for STR at the surface of SNPs/GO/SPE in the investigated potential range.

To reconfirm the CV experiments, electrochemical impedance spectroscopy (EIS) experiments were performed also which are shown in Figure 3B, with plots of the modified electrodes after each modification step. It is clearly noticeable that the bare SPE exhibits a small charge transfer resistance, but for the GO modified electrode, the Rct significantly increased to 517 Ω. Modification with OB causes a decrease in resistance compared to the value for GO/SPE.

**Figure 4:**
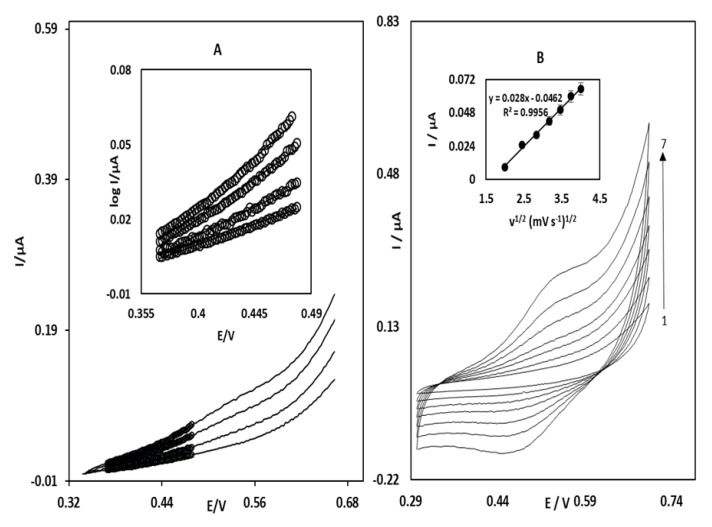
Linear sweep voltammograms of OB/SNPs/GO/SPE in 0.l M phosphate buffer solution pH = 7.0 containing 0.1 mM STR at different scan rates (The numbers 1–7 correspond to 4–16 mV·s^−1^). Inset B shows variation of the electrocatalytic current versus the square root of the scan rate.

The cyclic voltammograms of a 0.10 mM STR solution at different scan rate potentials are presented in Figure 4. The inset of Figure 4 indicates the linearity of the plot of the catalytic peak current versus the square root of the scan rate potentials, implying that the reaction is limited by diffusion. These results imply that the oxidation reaction of STR by OB involves a catalytic reaction (*E_r_C'_i_*). A theoretical model can be used to calculate the catalytic rate constant for *E_r_C′_i_* mechanisms. A relationship between the substrate concentration and the peak current for the case of a slow scan rate, *v*, and a large catalytic rate constant, *k′*, is constructed based on the theoretical model of Andrieux and Saveant [26] for such a mechanism. Using this model, and in particular Figure 4 of this work, we calculate an average value for *k*′ of 8.1 ± 0.07 cm s^−1^. The parameter *k′* is the catalytic rate constant between OB/SNPs/GO/SPE and STR. Thus, this value of *k′* defines a good catalytic characteristic for the oxidation of STR at OB/SNPs/GO/SPE.

According to the following equation for totally irreversible diffusion-controlled processes, the slope of the *I_p_* versus *v*^1/2^ plot can be used to obtain the number of electrons in the overall reaction (Figure 4, inset A) [27].

*I_p_* = 3.01 × 10^5^n[(1 − *α*)*n_α_*]^1/2^*AC_b_D*^1/2^*V*^1/2^ (4)

Using the obtained values of (1 − *α*) *n_α_* = 1.02 and *D* = 8.33 × 10^−6^ cm^2^·s^−1^ from the chronoamperometry method, the total number of electrons in the anodic oxidation of STR is estimated as *n* = 2. The linear sweep voltammograms of OB/SNPs/GO/SPE in a 0.1 M phosphate buffer is used to obtain information on the rate-determining step, using points of the Tafel region of the linear sweep voltammograms in Figure 4, and the anodic Tafel plots were drawn (inset B of Figure 4). The value of Tafel slope *b* = (1 − *α*)*n_α_ F*/2.3*RT* for STR indicates that this process is one-electron transfer for the rate-determining step, and the predicted mean charge transfer coefficient is α = 0.32 for STR. Furthermore, the exchange current density, *J*_0_, can be calculated from the intercept of the Tafel plots and geometric area. The obtained average value of *J*_0_ is 3.35 μAcm^−2^ for the oxidation of STR at the surface of modified electrode. This parameter shows the exchange current between the electroactive agents on the surface unite in equilibrium potential [28]. Therefore, the higher value of *J*_0_ indicates that charge transfer between the analyte and electrode surface is significant and displays a higher rate.

### 3.3. Chronoamperometric Studies of STR Oxidation at OB/SNPs/GO/SPE

**Figure 5:**
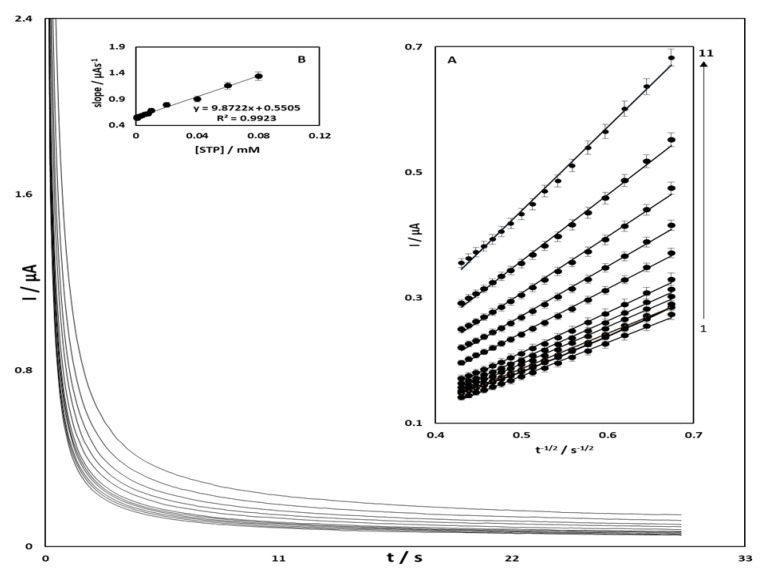
Chronoamperometric response of the OB/SNPs/GO/SPE in 0.1 M phosphate buffer solution pH = 7.0 at potential step of 570 mV for different concentrations of STR. The numbers 1–11 correspond to 0.001, 0.002, 0.004, 0.006, 0.008, 0.01, 0.02, 0.04, 0.06, 0.08, and 0.1 mM of STR, respectively. Insets: (**A**) plots of *I* versus *t^−1/2^* obtained from the Chronoamperograms and (**B**) plot of the straight lines against the STR concentration.

The electrocatalytic oxidation of streptomycin at OB/SNPs/GO/SPE surface was also studied by a chronoamperometry method. Chronoamperograms were obtained at different concentrations of streptomycin at a potential step of 570 mV (Figure 5). For an electroactive material (STR in this case) with an evident diffusion coefficient, *D_app_*, the current related to the electrochemical reaction (under diffusion control), is defined by the Cottrell equation:

*I* = *nFAD_app_*^1/2^*C*/*π*^1/2^*t*^1/2^ (5)

where *D_app_* is the recognizable diffusion coefficient and *C* is the bulk concentration of the analyte. The plots of *I* versus *t*^−1/2^ are shown in Figure 5, which is compared to different concentrations of STR. The obtained straight lines slopes were then plotted vs. the STR concentration (Figure 5). From the slope (9.87 µA s^1/2^ mmol L^−1^) and use of the Cottrell equation [29], an apparent diffusion coefficient (*D_app_*) of STR was calculated to be 8.33 × 10^−6^ cm^2^ s^−1^.

### 3.4. Simultaneous Determination of STR and OTC

**Figure 6:**
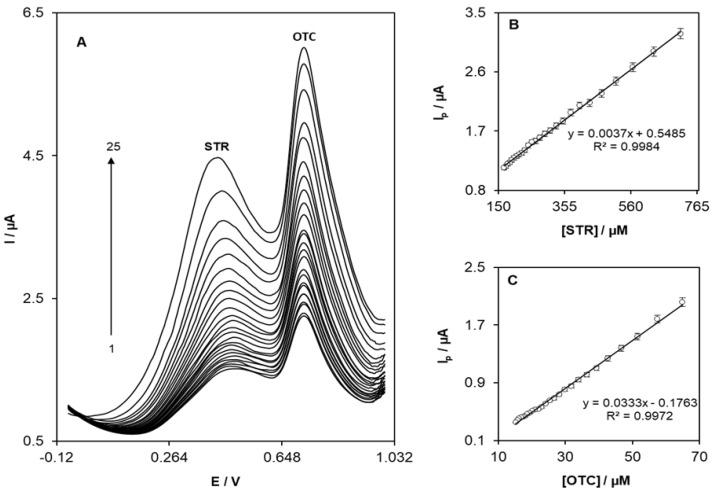
(**A**) Differential pulse voltammograms of OB/SNPs/GO/SPE in a 0.1M phosphate buffer solution pH = 7.0 containing different concentrations of STR and OTC. Numbers 1–25 correspond to (00.0–700.0 µM STR and 10.0–70.0 µM OTC. Insets: (**B**,**C**) show the plots of the electrocatalytic peak current as a function of STR and OTC concentrations, respectively.

Experiments were performed using OB/SNPs/GO/SPE at different concentrations of STR and OTC. Figure 6 shows the DPVs obtained with increasing concentrations of STR in the presence of different concentrations of OTC, and the inset A of (Figure 6) shows the differential pulse voltammetry plots of a combination of 0.1 mM STR and 0.01 mM OTC at OB/SNPs/GO/SPE. In this case, at the potentials of 540 and 740 mV two notable anodic peaks were observed, which correspond to the oxidation of STR and OTC, respectively. Furthermore, due to the addition of STR and OTC concentrations, significant increases in peak currents were detected. Accordingly, an effective oxidation reaction of the STR at OB/SNPs/GO/SPE is observed. The dependence of DPV peak currents on the STR and OTC concentration are shown in inset B and C of Figure 6, respectively, together with the linear relationship of the obtained peak current with increasing concentration of STR and OTC across the explored range.

**Figure 7:**
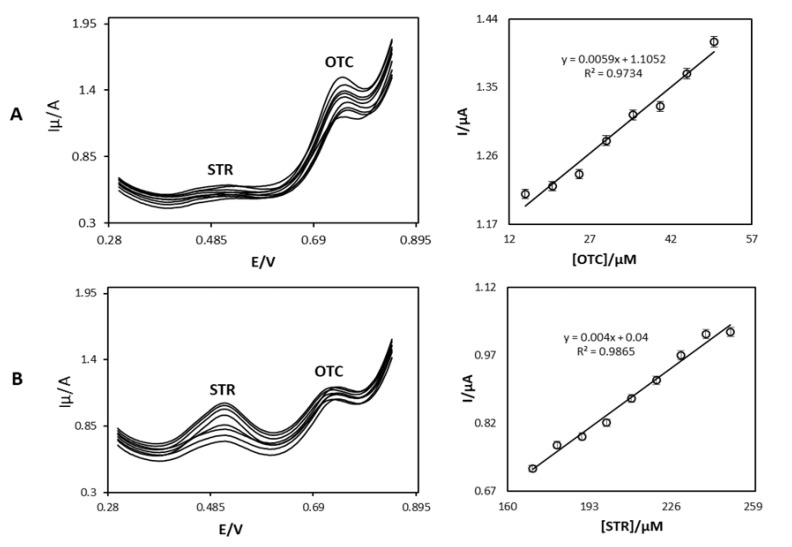
(**A**) Differential pulse voltammograms of OB/SNPs/GO/SPE in a 0.1 M phosphate buffer solution pH = 7.0 containing 15.0 µM OTC and different concentrations of STR. Numbers 1–9 correspond to 170.0, 180.0, 190.0, 200.0, 210.0, 220.0, 230.0, 240.0, and 250.0 µM STR, respectively. Inset shows the plot of the electrocatalytic peak current as a function of STR concentration within the 170.0–250.0 µM range. (**B**) Differential pulse voltammograms of OB/SNPs/GO/SPE in a 0.1 M phosphate buffer solution pH = 7.0 containing 170.0 µM STR and different concentrations of OTC. Numbers 1–8 correspond to 15.0, 20.0, 25.0, 30.0, 35.0, 40.0, 45.0, and 50.0 µM OTC, respectively. Inset shows the plot of the electrocatalytic peak current as a function of OTC concentration within the 15.0–50.0 µM range.

An attempt was also made at the determination of STR in the presence of OTC by using an OB/SNPs/GO/SPE sensor. Figure 7 shows the differential pulse voltammetric responses which were obtained by changing either the STR (Figure 7A) or OTC (Figure 7B) concentration, while the concentration of the other compound was constant. As depicted (Figure 7A), the electrochemical response of STR in the presence of a constant concentration of OTC increases linearly with the increase of the STR concentration, while the response of OTC remains almost constant. Similarly, Figure 7B shows the DPVs obtained by increasing the concentrations of OTC in the presence of STR. From these results, it can be noted that the responses to STR and OTC at the OB/SNPs/GO/SPE surface are independent from each other. From this work, the use of OB/SNPs/GO/SPE for the determination of STR in the presence of OTC has clearly been demonstrated.

**Figure 8:**
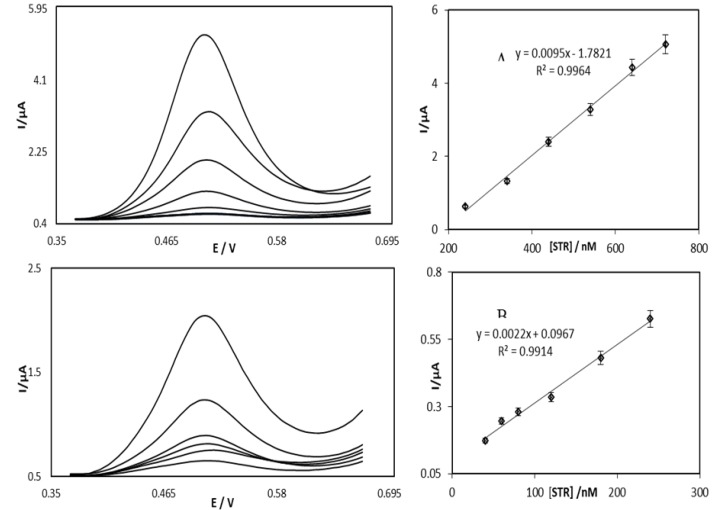
Differential pulse voltammograms of the GO/OB/SNP/SPE in 0.1 M phosphate buffer solution pH = 7.0 containing different concentrations of STR (from 0.36 to 0.67 V and peak potential of 0.54 V). The voltammograms correspond to STR. Inset A shows the plots of the electrocatalytic peak current as a function of STR concentration in the range of 0.4 to 240.0 nM and inset B is the peak current as a function of STR concentration in the range of 240.0 to 720.0 nM.

Determination of the linear ranges and limit-of-detection of STR were obtained using differential pulse voltammetry and the OB/SNPs/GO/SPE. (Due to the fact that DVP with respect to current sensitivity is much higher than cyclic voltammetry, the DPV method was used in this work.) Differential pulse voltammograms are shown in Figure 8, which were obtained from successive addition of STR at the step potential of 570 mV. The peak currents plot versus STR concentration shows two linear sections that rely on two different ranges of 0.4 to 240.0 nM and 240.0 to 720.0 nM at a potential step of 570 mV for STR. This is clearly indicated in the insets A and B of Figure 8. Based on reference [30] *Cm* is the lower limit of detection, which was found from the equation *Cm = 3s_bl_/m*, where *m* is the slope of the calibration curve (Figure 8) in the earlier linear range, and the standard deviation of the blank response (*s_bl_*) was found from fourteen repetitions of blank solution measurements. Consideration of the obtained data results in the value of 0.17 nM for the detection limit of STR. The relative standard deviation (*RSD*%) of OB/SNPs/GO/SPE was determined by 14 measurements of 0.1 mM STR solution and was found to be 2.9%.

In Table 1, a comparison of the obtained characteristics for STP in this research with other sensors was performed. As shown in Table 1, the introduced modified electrode displays higher responses in comparison with former reported modified electrodes.

## 4. Discussions

### 4.1. Analysis of Impedance Results

With respect to SNPs/GO/SPE, reduction of Rct occurred to 243 Ω, likely because of the higher surface area and excellent conductivity of SNPs. After immobilization of OB on the SNPs/GO/SPE, the Rct value decreased slightly to 401 Ω. These results clearly support the observations of CV experiments and reconfirmed the procedure employed for the modified electrodes.

### 4.2. Evaluation of Sensitivity and Stability of the Prepared Electrode 

The sensitivities of the OB/SNPs/GO/SPE towards the oxidation of STR in the absence and presence of OTC are 2.625 × 10^−1^ and 2.633 × 10^−1^ µA/µM, respectively. It is noteworthy that the sensitivity of the modified electrode to STR in the absence and presence of OTC is nearly the same, which shows that STR and OTC oxidation at the OB/SNPs/GO/SPE is independent of each other. This result confirms determination of STR in the presence of OTC is achieved without any form of interference.

In order to study the stability of the modified electrode, several OB/SNPs/GO/SPE have been produced with the above-described method. The modified electrodes were examined within a time range of 1 to 14 days as outlined above. The results showed that the modified electrodes have the best stability for use less than 7 days. After 7 days, the responses of the modified electrodes yield a more than 5% decrease. It is noteworthy that STR, OTC and their oxidation product(s) have no harsh effect on the modified electrode surface. Hence, OB/SNPs/GO/SPE was found to have several advantages such as fast response time, a good detection limit, a wide linear range and almost a stable response for the determination of STR and OTC.

### 4.3. Application

The application of the introduced electrochemical sensor for the determination of STR in real samples was examined by determining the concentration of the molecule in two milk samples. The preparation of the sample involved dilution of 3 mL of fresh milk to 10 mL with a 0.1 M phosphate buffer solution pH = 7.0. Next, the addition of certain amounts of STR was performed and the responses of the OB/SNPs/GO/SPE determined via DPV measurement. The results are shown in Table 2. It is clear that OB/SNPs/GO/SPE can be successfully used for STR determination in different milk samples. Therefore, the components of the milk matrix do not interfere with determination of STR as determined by the proposed sensor.

### 4.4. Reliability and Interference study of Proposed Nanosensor

The reliability of the proposed nanosensor was evaluated by comparing the results with those obtained from that declared in the label of the dairy products and LC/MS standard method to our method in order to quantify STR in the same sample. The concentrations of STR in the milk samples were found to be 19.0 ± 0.5 µM and 62.0 ± 1.2 µM by the present voltammetric method, which is in close agreement with the values of 21.1 ± 0.2 µM and 63.3 ± 0.8 µM obtained by the standard method for STR and declared in the label of the milk package. Based on the *t* test, it can be concluded that there is no evidence of systematic difference between the results obtained by two methods. This suggests that the detection procedures have been free from any interference from components of the sample matrix.

## 5. Conclusions

In this work, we introduce a very simple, fast, and precise electrochemical technique for the determination of both STR and OTC. The obtained results indicate that OB/SNPs/GO/SPE was effective in detecting STR and OTC separately and in the determination of both components in the mixture. The OB/SNPs/GO/SPE electrode revealed excellent electrocatalytic properties for STR oxidation in a phosphate buffer solution pH = 7.0. Moreover, the modified electrode played a role as an excellent agent for oxidation of OTC in comparison to GO/SPE. The cyclic voltammetric results show that STR displays the characteristics of an E_r_C'_i_ catalytic mechanism. In DPV measurements, the separation of the STR and OTC oxidation peak potentials at the surface of OB/SNPs/GO/SPE electrode is around 200 mV. Determination of STR in the presence of OTC in milk samples has been achieved with satisfactory results by using OB/SNPs/GO/SPE.

## Figures and Tables

**Table 1:** Comparison of the present method with other analytical methods.

Method	Linear Range (μM)	LOD (μM)	Matrices	Ref
LC/MS	-	3, 0.6	Milk, Honey	[31]
LC/MS	-	4.7	Honey	[32]
Electrochemistry	-	14.1, 15.3	Milk, Serum	[33]
Electrochemistry	8.6 × 10^−3^–3.44 × 10^−2^	1.7 × 10^−2^	Food	[4]
Electrochemistry	1.0–33.8 33.8–81.7	1.0	Honey	[34]
Electrochemistry	860.0–8.60.0 × 10^6^	5.0 × 10^9^	Muscle/Kidney/milk/Honey	[35]
Electrochemistry		7.0 × 10^6^	Milk	[36]
Electrochemistry	1.0–1.0 × 10^3^	1.5 × 10^−3^	Injection Sample Solution	[37]
Electrochemistry	4 × 10^−4^–2.4 × 10^−1^ 2.4 × 10^−1^–7.2 × 10^−1^	0.17 × 10^−3^	Milk	This Work

**Table 2:** Determination and recovery results of streptomycin in two different milk samples.

Sample	Added (µM)	Found (µM)	RSD (%)	Recovery (%)
**Milk1**	0	19	3.42	-
	10	29.2	3.26	100.68
	20	38.7	3.08	99.23
	30	49.2	3.16	100.40
**Milk2**	0	62	3.49	-
	10	72.4	4.39	100.55
	20	81.9	3.03	99.87
	30	92.5	4.18	100.54
